# Brief intervervention for a metabolic syndrome in psychiatric outpatients with severe/persistent mental illness: A prospective study

**DOI:** 10.1192/j.eurpsy.2023.1915

**Published:** 2023-07-19

**Authors:** M. Escarti, C. Alonso, L. Fernandez, O. Ribera, A. Beato, R. Cozar, G. Selva

**Affiliations:** Psychiatry, Clinic Hospital Valencia, Valencia, Spain

## Abstract

**Introduction:**

Metabolic syndrome and other cardiovascular risk factors are highly prevalent in people with mental severe illness (Sun & Jang, 2020).Metabolic disorders in people with schizophrenia increase their risk of developing cardiovascular disease, consequently reducing their life expectancy by approximately 10 to 25 years (Heald et al., 2017)In part these cardio-metabolic risk factors are attributable to unhealthy lifestyle, including poor diet and sedentary behaviour.Lifestyle interventions (diet, increased physical activity) are the first-line treatments to decrease that risk.

**Objectives:**

Our objective is to carry out a prospective study on the application of a program of healthy habits in outpatients unit

**Methods:**

Patients with mental severe illness were recruited at a mental health center in the Hospital Clinic of Valencia. Inclusion criteria: age from 18 to 65 years and diagnosis of severe/persistent mental illness Exclusion criteria: acute illness, were not understanding Spanish, not be able to read and understand questionnaries. We included following data: sociodemographic data and aspects of the health behaviors, anthropometric measurements and analytical with hemogram and biochemistry pre and post-intervention. All subjects gave informed consent for participation in the study.

**Results:**

We included 12 patients, but only 9 completed the full program. Average baseline data suggests that participants were at increased health risk when entering the program.At the end of the program, differences were observed: a reduction in glucose profile, a reduction of an average of 3.33 kg from the initial weight and a reduction of 10 points in blood pressure.

**Conclusions:**

This real world pilot trial evaluate of a health promotion intervention targeting physical activity and healthy eating in mental health care using a specific programme.

**Disclosure of Interest:**

None Declared

